# Apple fruitlet physiological characteristics and their influence on diffuse visible/near-infrared reflectance spectroscopy

**DOI:** 10.1093/aob/mcaf124

**Published:** 2025-06-19

**Authors:** J E Larson, T Zuber, T M Kon

**Affiliations:** Department of Plants, Soils, and Climate, Utah State University, Logan, UT 84322, USA; Department of Horticultural Sciences, North Carolina State University, Mountain Horticultural Crops Research and Extension Center, Mills River, NC 28759, USA; Department of Horticultural Sciences, North Carolina State University, Mountain Horticultural Crops Research and Extension Center, Mills River, NC 28759, USA

**Keywords:** Apple, *Malus × domestica* L. Borkh, chlorophyll, trichome, reflectance spectroscopy

## Abstract

**Background and Aims:**

The recent rise in digital technologies to inform agronomic practices has necessitated further understanding of how plant physiological characteristics affect light reflectance measurements that these technologies employ. A young apple fruitlet undergoes rapid changes in size, chlorophyll content and trichome density as it develops. Our objective here was to characterize these changes in ‘Fuji’ and ‘Honeycrisp’ fruit to understand how those changes affect diffuse spectral reflectance.

**Methods:**

Visible/near-infrared reflectance spectra were captured with a portable visible and near-infrared spectrometer on individual fruit from 20 to 36 d after full bloom. From these reflectance spectra principal component analysis (PCA) was performed and vegetation indices were calculated. A method was developed to quantify trichome density using image analysis. Water content, trichome density and chlorophyll concentration were determined for each fruitlet. Regression analysis and correlation coefficients were calculated to understand how these measures relate during fruitlet development.

**Key Results:**

PCA revealed the first principal component contributed to 86.56 % of the variation in the reflectance data. Along this component there was a gradient of fruit size. These size effects led to lower reflectance at wavelengths associated with chlorophyll and water absorption. Chlorophyll concentration and trichome density decreased with increasing fruit size. The total amount of chlorophyll in the fruit increased as fruit grew larger. Total chlorophyll content had a stronger relationship with fruit size than with trichome density.

**Conclusions:**

Fruit size and in turn total chlorophyll content had the greatest effects on reflectance spectra. When measuring reflectance with a portable visible and near-infrared spectrometer that has a fixed lens size, the amount of tissue covering that lens is a major influence on the reflectance spectra. There were differences in chlorophyll content between the two apple cultivars studied that may have larger implications for the photosynthetic capacity of fruit.

## INTRODUCTION

The use of technology to inform agronomic practices has grown exponentially in recent years. These applications are wide ranging, including but not limited to: detecting water stress (Govender *et al*., 2009), nutrient deficiency (Watt *et al*., 2019; Burns *et al*., 2022) and yield prediction (Prasad *et al*., 2006). Remote sensing and spectroscopy are two of these technologies, both relying on the measurement of light interaction with plant material. Physiological characteristics of the plant influence this light interaction. Measurement of red-light is used in many remote sensing and close-range applications. The finding that chlorophyll content of leaves is closely related to nitrogen content (Wood *et al*., 1993) and that chlorophyll absorbs red-light allowed for these technologies to detect nitrogen deficiencies. These studies underscore the importance of understanding the relationship between plant physiological characteristics and measurement of light reflectance with emerging technologies to develop applications in agriculture.

In apples, the majority of image technologies have focused on detecting and counting flowers (Dias *et al*., 2018; Sun *et al*., 2021) and fruit (Ji *et al*., 2012; Gonzalez Nieto *et al*., 2023) in the canopy to aid in crop load management decisions and yield prediction. The intentional removal (thinning) of fruit to reach a sustainable crop load is one of the most important management decisions faced by growers. The application of plant bioregulators (chemical thinners) to thin fruit is the most efficient method that growers have. The response to these applications is often erratic (Lakso *et al*., 1999), necessitating multiple applications that must occur in the brief 3- to 4-week period in the spring when trees are susceptible to chemical thinners (Byers *et al*., 1990). Tools to predict the efficacy of these applications help growers know whether they should make another chemical thinner application while trees are still susceptible. Thermal imaging (Populin *et al*., 2022) and reflectance spectroscopy (Orlova *et al*., 2020*a*; Larson and Kon, 2023) are two technologies that have been used to detect which fruit will abscise following application of a chemical thinner. Elucidating the role that these physiological characteristics play in measuring light reflectance will guide development of models to predict chemical thinner efficacy that have applicability across cultivars, growing regions and seasons.

To predict chemical thinner efficacy, reflectance spectroscopy relies on differences in the amount of light reflected at various wavelengths between fruitlets that ultimately persist and abscise. Both Orlova *et al*. (2020*a*) and Larson and Kon (2023) used portable handheld spectrometers that measured reflectance of visible (Vis; ∼400–700 nm) and near-infrared (NIR; ∼700–1500 nm) light. Persisting fruitlets consistently exhibit lower reflectance of red-light (∼600 nm) than abscising fruitlets (Orlova *et al*., 2020*b*; Larson *et al*., 2023). Chlorophyll is the main driver of red-light absorbance (Sims and Gamon, 2002; Merzlyak *et al*., 2003). Orlova *et al*. (2020*b*) hypothesized that lower trichome density of persisting fruitlets allowed for more light to be absorbed by chlorophyll and led to lower reflectance in this region. This was supported by their findings that reflectance from 400 to 1100 nm was lower for fruit that had trichomes removed than for intact fruit (Orlova *et al*., 2020*b*). Larson *et al*. (2023) proposed that the total chlorophyll content of the fruitlet also contributed to this trend as fruit that were predicted to persist had higher chlorophyll content than those predicted to abscise. Light of between 500 and 700 nm has been found to travel ∼4 mm into mature apple fruit, while penetration depth is ∼3 mm from 900 to 1300 nm (Lammertyn *et al*., 2000); to our knowledge, it is unknown how far light travels into young fruit. This means that physiological characteristics on the surface and interior of the fruit, their interaction with light, and incident light around the spectrometer all affect reflectance spectroscopy.

The trichomes present on the surface of fruit are non-glandular, unicellular and ∼1 mm long (Konarska, 2014). In early fruit development, Orlova *et al*. (2020*b*) observed that trichomes were denser on smaller fruitlets (∼4 mm in diameter) compared to larger fruit (∼9 mm in diameter) and that this decrease in density was greatest at the centre of the fruit compared to the calyx or pedicel ends. Orlova *et al*. (2020*b*) speculated that this decrease in density occurs because following bloom the trichome number stays constant, so density decreases as fruit expand. The positional effect of trichome density aligns with fruit growth patterns as fruit growth rate is least at the calyx end (Pratt, 1988). In addition to trichomes, waxes are present on the surface of fruit and could affect light reflectance. Faust and Shear (1972) found that the size and structure of fruit surface wax varied among cultivars. Cuticular wax deposits ranged from 2 to 6 µm in depth with wax crystal morphology being upright on ‘York Imperial’ or flattened on ‘Golden Delicious’ at 40 d after full bloom (DAFB). Together, trichomes and wax deposits represent the first components of a fruit to interact with light.

Chlorophyll is present in the peel of apples throughout much of fruit development, but degrades as fruit ripen (Blanke and Lenz, 1989). Early in the growing season, chlorophyll is present in cells throughout the cortex of the fruit, but as these cells expand chlorophyll concentration decreases (Larson *et al*., 2023). This loss is rapid as chlorophyll concentration decreased from ∼850 to 450 µg g^−1^ dry weight within a span of 10 d in early fruit development (Larson *et al*., 2023). Apple fruit rely mostly on imported carbohydrates for sustained growth (Blanke and Lenz, 1989; Jing and Malladi, 2020). However, the ability of a young fruitlet to photosynthesize may be important for its continued development when carbohydrates become limiting (Larson *et al*., 2022). In turn, the amount of chlorophyll in a fruit may influence how susceptible it is to a chemical thinner.

Apart from plant pigments, water is the other main constituent that affects reflectance spectroscopy in the range of wavelengths investigated in the current study (303–1098 nm). There is a decrease in the spectral reflectance at ∼960 nm that is due to water absorbance (Peñuelas *et al*., 1993). Water plays an important role in apple fruit abscission early in the season. Fruit that ultimately abscise after application of a chemical thinner have a lower rate of water uptake (Ward and Marini, 1999) and water content (Larson *et al*., 2023).

The objective of this study was to determine the relative contribution of physiological characteristics – trichome density, chlorophyll content and water content – on the light reflectance of apples throughout early fruit development. This was explored on two apple cultivars with differing levels of sensitivity to chemical thinners, one that is known to be easy-to-thin – ‘Honeycrisp’ – and one that is hard-to-thin – ‘Fuji’ – to determine how these relationships can affect the prediction of thinner efficacy with reflectance spectroscopy across cultivars.

## MATERIALS AND METHODS

### Plant material and collection

This experiment was conducted in 2023 with apple fruit from two mature orchards in North Carolina, USA. The selected cultivars were 7-year-old ‘Cameron Select Honeycrisp’/‘Malling 9’ (Waynesville, NC, USA) and 5-year-old ‘DT2 Aztec Fuji®’/‘Geneva 41’ (Mills River, NC, USA). There were two collection dates for each cultivar – ‘Fuji’ at 23 and 36 DAFB with *n* = 47 and 45 fruit, respectively; and ‘Honeycrisp’ at 20 and 29 DAFB with *n* = 36 and 29 fruit, respectively; in total *n* = 158 fruit.

Data were taken on each individual fruit in ten total spurs for each cultivar and collection date. Spurs were collected throughout the exterior of the canopy. Trees for both cultivars were semi-dwarfed and had a uniform light environment throughout the canopy. Spurs were removed from the tree at the base of the spur, with the woody tissue still intact, and immediately placed into water-saturated floral foam (WetFōM; FloraCraft, Ludington, MI, USA) to maintain hydration of tissue at room temperature in the laboratory. All fruit collected from spurs followed the same steps for analysis: (1) capture reflectance spectra and fresh weight of intact fruit, (2) remove a longitudinal strip of trichomes for quantification, (3) remove all trichomes from fruit and re-capture reflectance spectra, and (4) freeze fruit for later chlorophyll quantification. These four steps are explained in the subsequent sections.

### Reflectance spectra and spectral indices

Reflectance spectra were taken with a portable visible and near-infrared spectrometer (Felix F-750; Felix Instruments, Camas, WA, USA). This spectrometer has a spectral resolution of 3.3 nm and a range from 303 to 1098 nm. This spectrometer takes four scans in total for each reading: ‘open’ (lamp on/off) reading of the sample and then a ‘closed/dark’ (lamp on/off) reading with a shutter covering the spectrometer. The resulting reflectance is the difference between the shutter open lamp on and shutter open lamp off. The closed scans are used to correct the open scans in a reading. To direct more light from the lamp to the sample, a cone produced by the manufacturer was placed around the lens. Reflectance of each fruit was measured individually with the calyx end of the fruit placed flush with the lens of the spectrometer. Each fruit was manually held in place with light pressure to maintain contact of the calyx end with the lens throughout measurement. Reflectance spectra were captured for the intact fruit and after all trichomes were removed.

Two spectral reflectance indices were calculated from the reflectance data for the intact fruit: a chlorophyll concentration index (CCI) following Gitelson *et al*. (2003) and a plant water index (PWI) following Peñuelas *et al*. (1993), as summarized in eqns (1) and (2), respectively, below:


(1) $$\mathrm{CCI}=R_{522-579}/R_{640-700}$$

(2) $$\mathrm{PWI}=R_{950-970}/R_{890-900}$$

In eqns (1) and (2), *R* is the average reflectance over the indicated range of wavelengths. For example, *R*_950–970_ is the average reflectance from each wavelength between 950 and 970 nm.

### Trichome quantification

**Fig. 1.**
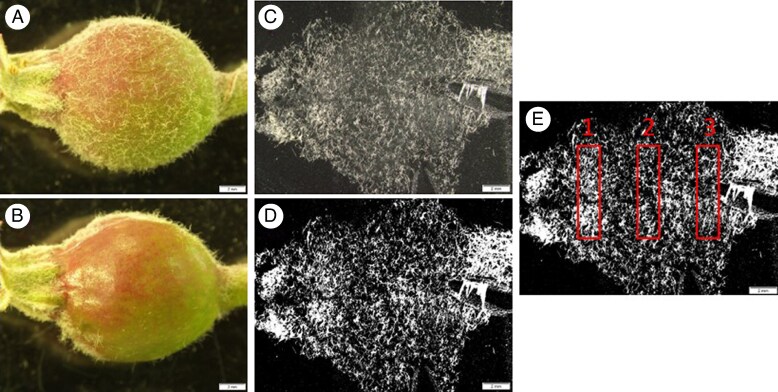
(A) Trichome density quantification of immature apple fruit. (B) Apple following removal of longitudinal strip of trichomes with tape. (C) Image of removed trichomes. The image is then converted to a binary 8-bit image (D) where pixels with trichomes have an intensity of 1.0 and everything else has an intensity 0.0. (E) The percentage of pixels with an intensity of 1.0 are quantified in a 1.75 × 7.0 mm rectangle at three positions along the fruit: the calyx end (1), the middle of the fruit (2) and pedicel end (3). In all images the scale bar is 2.0 mm.

The following methodology was developed to quantify trichome density. First, a longitudinal section of each fruit extending from the base of the calyx to the base of the pedicel had a piece of black electrical tape (19.05 mm width; 3M Vinyl Electrical Tape; 3M, St. Paul, MN, USA) firmly pressed down to the targeted area and then rapidly peeled off to remove a section of trichomes. This longitudinal section of tape with removed trichomes was imaged with a stereo microscope (Olympus SZX16; Olympus-lifesciences, Shinjuku City, Japan) equipped with a mounted camera (Olympus DP22). This section was imaged with a total on-screen magnification of 23×. Each image was converted to an 8-bit, binary (black/white) image with a 1.33 aspect ratio in ImageJ (v.1.53t; National Institute of Health, Bethesda, MD, USA). Within each image three equidistant rectangular subsections each measuring 1.75 mm vertically by 7.00 mm horizontally (12.25 mm^2^; 4.43 × 10^−6^ mm^2^ per pixel) positioned near the calyx, centre and pedicel respectively were created (Fig. 1). In the binary image, the trichomes had a pixel intensity of 1, while the background had an intensity of 0. A threshold based on pixel intensity of the binary image was used for trichome quantification. Percentage coverage of trichomes in the total area of the rectangular subsection was quantified as the total number of pixels after the threshold compared to the number of pixels in the original image.

### Chlorophyll quantification

Chlorophyll was quantified following the methods of Larson *et al*. (2023) that adapted a method for plant pigment quantification by spectrophotometry from Wrolstad *et al*. (2005). Following storage at −20 °C, fruit samples were freeze dried (VirTis LyoTroll; SP Scientific, Warmister, PA, USA) for 7 d and dry matter content (g) of each fruit was recorded. A genogrinder (Genogrinder 2010; SPEX, Metuchen, NJ, USA) was used to grind each sample to a fine powder with two 9-mm steel balls. A 0.02-g subsample of ground tissue was used for chlorophyll quantification. First, 1.45 mL of 95 % ethanol was mixed with this subsample and vortexed for 2 min with two 5-mm steel balls (Mortexor Vortex; Benchmark Scientific, Edison, NJ, USA). This was then centrifuged for 10 min at 10 000 *g*_n_ and 4 °C (model 5417R; Eppendorf, Enfield, CT, USA). The resulting supernatant (∼1 mL) was then collected and transferred to a spectrophotometer cuvette. A benchtop spectrophotometer (model 2450; Shimadzu, Columbia, MD, USA) was then used to quantify absorbance (ABS) of the sample at 644.1, 648.6 and 750.0 nm. Using these ABS values, chlorophyll *a* (µg g^−1^ DW; eqn 3), chlorophyll *b* (µg g^−1^ DW; eqn 4) and total chlorophyll (µg g^−1^ DW; eqn 5) were quantified for each sample:


(3) $$\begin{array}{rl} \mathrm{Chlorophyll}\;a\;= & [13.36\;\times (\mathrm{AB}S_{664.1}-\;\mathrm{AB}S_{750})-\;5.19\;\\ & \times (\mathrm{AB}S_{648.6}-\;\mathrm{AB}S_{750})]\times \mathrm{DF} \end{array}$$

(4) $$\begin{array}{rl} \mathrm{Chlorophyll}\;b\;= & [27.43\;\times (\mathrm{AB}S_{648.6}-\;\mathrm{AB}S_{750})\\ & -\;5.19\;\times (\mathrm{AB}S_{664.1}-\;\mathrm{AB}S_{750})]\times \mathrm{DF} \end{array}$$

(5) Totalchlorophyll=chlorophylla+chlorophyllb

In the above equations, the dilution factor (DF) is the total amount of solvent used (1.45 mL).

### Data analyses

For each individual fruit collected between two measurement dates for both cultivars, we quantified: fresh weight (g); reflectance from 303 to 1098 nm (a.u.); trichome density near calyx, centre and fruit pedicel (%); dry weight (g); PWI (a.u.); CCI (a.u.); concentration of chlorophyll *a*, *b* and total (µg g^−1^ DW); water content (%); and amount of chlorophyll (µg). All data preparation and analysis was done in R (R Foundation for Statistical Computing, Vienna, Austria, R Core Team, 2022). Principal component analysis (PCA) was performed on the standardized reflectance spectra. For each cultivar, linear regression was done between fruit fresh weight by trichome density at each position of the fruit (calyx, centre, pedicel), and total chlorophyll concentration and amount. To detect differences between cultivars, at common weights, linear regression was done with the interaction between fruit weight and cultivar in the model. If this interaction term was significant then the slopes between the two cultivars differed. Second-order polynomial regression was conducted between CCI/PWI and fruit fresh weight. A Pearson correlation coefficient was determined for every combination of each measured response. PCA, regression and Pearson correlation coefficients were all computed with the stats package (R Core Team, 2022).

## RESULTS

### Fruit size and dry matter content

**Fig. 2.**
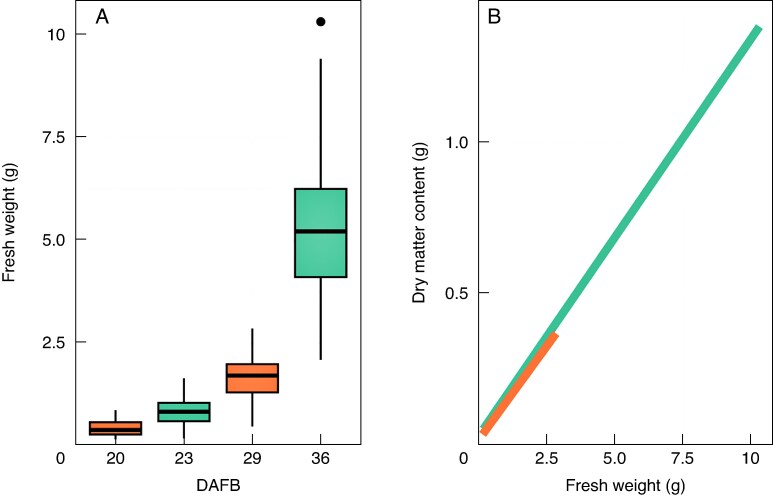
(A) ‘Fuji’ (teal) and ‘Honeycrisp’ (orange) fruit fresh weight (g) distribution by sampling date (days after full bloom; DAFB) and (B) linear regression between fresh weight and dry weight [g (‘Fuji’: *r*^2^ = 0.993, *P* < 0.001; ‘Honeycrisp’: *r*^2^ = 0.869, *P* < 0.001)] of apple fruit sampled in North Carolina, USA, in 2023. Shaded area is the confidence interval of the regression line. *N* = 90 (‘Fuji’); *N* = 65 (‘Honeycrisp’).

Fresh weight of collected fruit varied across cultivars with the staggered collection dates (Fig. 2A). On 20 DAFB, ‘Honeycrisp’ ranged from 0.17 to 0.88 g. On the first collection date for ‘Fuji’ (23 DAFB) the minimum fresh weight was 0.19 g and the maximum was 1.65 g. The second collection period for ‘Honeycrisp’ (29 DAFB) had a fresh weight range of 0.48–2.86 g. On 36 DAFB, the fresh weight of ‘Fuji’ ranged from 2.10 to 10.30 g.

Dry matter content was closely related to fruit fresh weight [‘Fuji’: *r*^2^ = 0.993, *P* < 0.001; ‘Honeycrisp’: *r*^2^ = 0.869, *P* < 0.001 (Fig. 2B)]. Between cultivars, dry matter content was similar among common fruit sizes (0.19–2.86 g). The rate of dry matter content accumulation with increasing fruit size was also similar, as the slope of the regression equation was 0.132 (*P* < 0.001) and 0.124 (*P* < 0.001) for ‘Fuji’ and ‘Honeycrisp’, respectively.

### Reflectance spectra

**Fig. 3.**
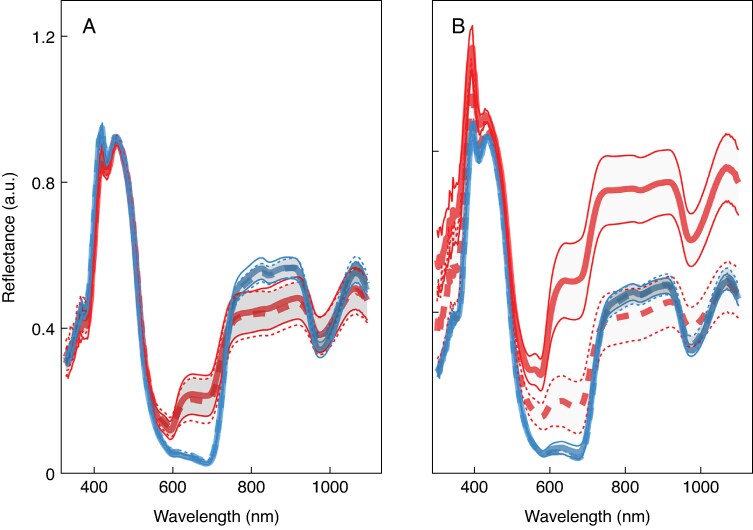
Mean reflectance (a.u. ± s.e.) from 303 to 1098 nm of ‘Fuji’ (A) and ‘Honeycrisp’ (B) apple fruit sampled in North Carolina, USA, in 2023. Red lines are fruit sampled on the first measurement date (‘Fuji’: 23 DAFB; ‘Honeycrisp’: 20 DAFB). Blue lines correspond to fruit from the second measurement date (‘Fuji’: 36 DAFB; ‘Honeycrisp’: 29 DAFB). Grey area indicates the mean reflectance ± s.d. at each measured wavelength. Dashed lines are for intact fruit and solid lines are the same fruit after trichome removal. Reflectance measured with a portable Vis/NIR spectrometer (Felix F750; Felix Instruments, Camas, WA, USA) with a resolution of 3 nm. *N* = 90 (‘Fuji’); *N* = 65 (‘Honeycrisp’).

The shape of spectral reflectance curves was similar across collection dates, cultivars and with/without trichomes (Fig. 3). There were four areas in the curves where reflectance values change markedly. First, reflectance increases at ∼375 nm and peaks at ∼425 nm. There is then a rapid decrease in reflectance to a minimum from 560 to 640 nm. At this minimum there is a noticeable difference between collection dates for both cultivars. On the earlier collection date (20 and 23 DAFB for ‘Honeycrisp’ and ‘Fuji’, respectively), there is an increase in reflectance at ∼600 nm and plateauing before the curve begins to increase at ∼640 nm. For ‘Honeycrisp’ this increase was much more pronounced when fruit were measured without trichomes than on intact fruit. On the second collection date (29 and 36 DAFB for ‘Honeycrisp’ and ‘Fuji’, respectively), this uptick in reflectance does not occur and reflectance stays relatively constant at this minimum from 560 to 640 nm. Reflectance rapidly increases again, and plateaus at ∼750 nm. Finally, there is a dip in reflectance at ∼975 nm.

While there were few differences in the shape of the reflectance curves, there were differences in the magnitude of reflectance between measurement dates (Fig. 3). Reflectance between 560 and 640 nm was lower on the second collection date compared to the first for both cultivars. At the plateau in the NIR range (750–900 nm), reflectance was greater for intact fruits on the second collection date compared to the first for both cultivars. However, at ∼975 nm the dip in reflectance is lower on the second collection date than the first. Only for ‘Honeycrisp’ collected on 20 DAFB did removal of trichomes alter reflectance. There, intact fruit had lower reflectance from 303 to ∼375 nm and from ∼540 to 1098 nm than compared to fruit after trichomes were removed.

**Fig. 4.**
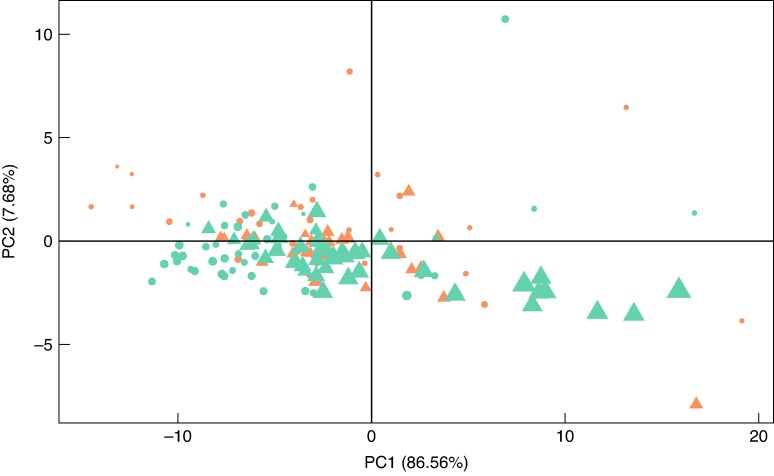
Principal component analysis of reflectance values (*n* = 155) at measured wavelengths (303–1098 nm; 266 wavelengths) of apple fruit sampled in North Carolina, USA, in 2023. The first principal component (PC1) accounts for 86.56 % of the variance and second principal component (PC2) accounts for 7.86 %. Together the first two principal components account for 94.42 % of the variance in the data. Size of observation corresponds to fresh weight of fruit (g). Orange: ‘Honeycrisp’ (*n* = 65), teal: ‘Fuji’ (*n* = 90). Circle: first sampling date (‘Fuji’: 23 DAFB, *n* = 47; ‘Honeycrisp’: 20 DAFB, *n* = 36); triangle: second sampling date (‘Fuji’: 36 DAFB, *n* = 43; ‘Honeycrisp’: 29 DAFB, *n* = 29).

More than 94 % of the total variance in the reflectance data was explained by the first two principal components (PC) in PCA (Fig. 4). PC1 explained 86.56 % of the variance and PC2 explained 7.68 % of the remaining variance. Fruit size appeared to be associated with this variation as there is a shift along PC1, as smaller fruit fall on the negative end and larger fruit on the positive end of PC1. The only clear clustering along PC2 was where there was a separation between cultivars from the first collection date (23 and 20 DAFB for ‘Fuji’ and ‘Honeycrisp’, respectively).

### Trichome density

**Fig. 5.**
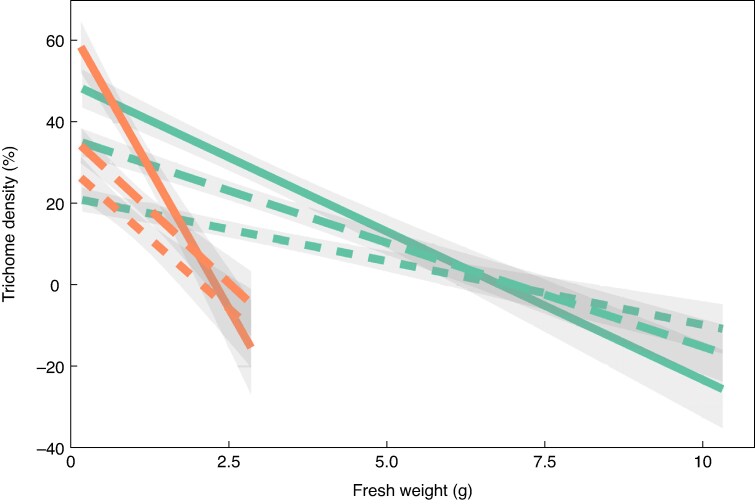
Linear regression between trichome density (%) and fresh weight (g) of ‘Fuji’ (teal) and ‘Honeycrisp’ (orange) apple fruit sampled in 2023 in North Carolina, USA. Shaded area is the 95 % confidence interval of the regression line. Trichome density was measured at the calyx end [solid line (‘Fuji’: *r*^2^ = 0.608; *P* < 0.001; ‘Honeycrisp’: *r*^2^ = 0.586; *P* < 0.001)], centre [dotted line (‘Fuji’: *r*^2^ = 0.419, *P* < 0.001; ‘Honeycrisp’: *r*^2^ = 0.339, *P* < 0.001)] and pedicel end [dashed line (‘Fuji’: *r*^2^ = 0.589, *P* < 0.001; ‘Honeycrisp’: *r*^2^ = 0.451, *P* < 0.001) of the fruit. *N* = 90 (‘Fuji’); *N* = 65 (‘Honeycrisp’).

For both cultivars, trichome density at the calyx, centre and pedicel end decreased as fruit size increased (Fig. 5). The decrease was more rapid for ‘Honeycrisp’ than for ‘Fuji’. Trichome density was greatest at the calyx end of the fruit for the smallest fruit but showed the most rapid decrease in trichome density during fruit development compared to the centre or pedicel end. Due to the rapid decrease in trichome density at the calyx end, there is an inflection point after which trichome density is lowest at the calyx end than the centre or pedicel end. For ‘Fuji’ this inflection point occurs at ∼6.5 g compared to ∼2.5 g for ‘Honeycrisp’. Fruit size was most directly related to trichome density at the calyx end (‘Fuji’: *r*^2^ = 0.608, *P* < 0.001; ‘Honeycrisp’: *r*^2^ = 0.586, *P* < 0.001) compared to the pedicel end (‘Fuji’: *r*^2^ = 0.589, *P* < 0.001; ‘Honeycrisp’: *r*^2^ = 0.451, *P* < 0.001) or centre (‘Fuji’: *r*^2^ = 0.419, *P* < 0.001; ‘Honeycrisp’: *r*^2^ = 0.339, *P* < 0.001) of the fruit.

The trend in the correlations between trichome density at the three sampled locations (calyx end, centre and pedicel end of fruit) and the two measured reflectance indices (CCI and PWI) varied between cultivars (Table 1). For ‘Honeycrisp’, trichome density at the calyx end showed the highest correlation coefficient with CCI [−0.565 (*P* < 0.001)] and PWI [0.733 (*P* < 0.001)]. These correlation coefficients were lowest for trichome density at the centre of the fruit [CCI: −0.477 (*P* < 0.001); PWI: 0.556 (*P* < 0.001)] and intermediary with pedicel-end trichome density [CCI: −0.547 (*P* < 0.001); PWI: 0.587 (*P* < 0.001)]. With ‘Fuji’ there was no clear trend between the two indices. CCI had the greatest correlation coefficient with trichome density at the centre of the fruit [−0.577 (*P* < 0.001)], followed by the calyx end [−0.554 (*P* < 0.001)], then the pedicel end [−0.488 (*P* < 0.001)]. For PWI, trichome density at the pedicel end had the highest correlation coefficient [0.706 (*P* < 0.001)], then the calyx end [0.691 (*P <* 0.001)] and finally the centre of the fruit [0.657 (*P* < 0.001)].

**Table 1.** Pearson correlation coefficients between measured responses of ‘Fuji’ (*n* = 90) or ‘Honeycrisp’ (*n* = 65) apple fruit sampled from 20 to 36 d after full bloom in North Carolina, USA, in 2023.

	Fruit fresh weight (g)		Chlorophyll concentration index (a.u.)		Plant water concentration index (a.u.)	
	Fuji	HC	Fuji	HC	Fuji	HC
Calyx end trichome density (%)	−0.779***	−0.765***	−0.554***	−0.565***	0.691***	0.733***
Centre of fruit trichome density (%)	−0.648***	−0.582***	−0.577***	−0.477***	0.657***	0.556***
Pedicel end trichome density (%)	−0.767***	−0.672***	−0.488***	−0.547***	0.706***	0.587***
Chlorophyll *a* concentration (µg g^−1^ DW)	−0.868***	−0.791***	−0.433***	−0.463***	0.673***	0.648***
Chlorophyll *b* concentration (µg g^−1^ DW)	−0.871***	−0.524***	−0.429***	−0.233	0.672***	0.356**
Chlorophyll concentration (µg g^−1^ DW)	−0.87***	−0.746***	−0.432***	−0.419***	0.673***	0.594***
Total chlorophyll content (µg)	0.944***	0.906***	0.575***	0.565***	−0.741***	−0.764***
Water content (%)	0.393***	0.145	0.16	0.127	−0.258*	−0.09
Fruit fresh weight (g)	–	–	0.423***	0.591***	−0.664***	−0.827***

HC = ‘Honeycrisp’. Asterisks indicate *P-*values for the correlation coefficients (*P < 0.05, **P < 0.01, ***P < 0.001).

### Chlorophyll content and chlorophyll concentration index

**Fig. 6.**
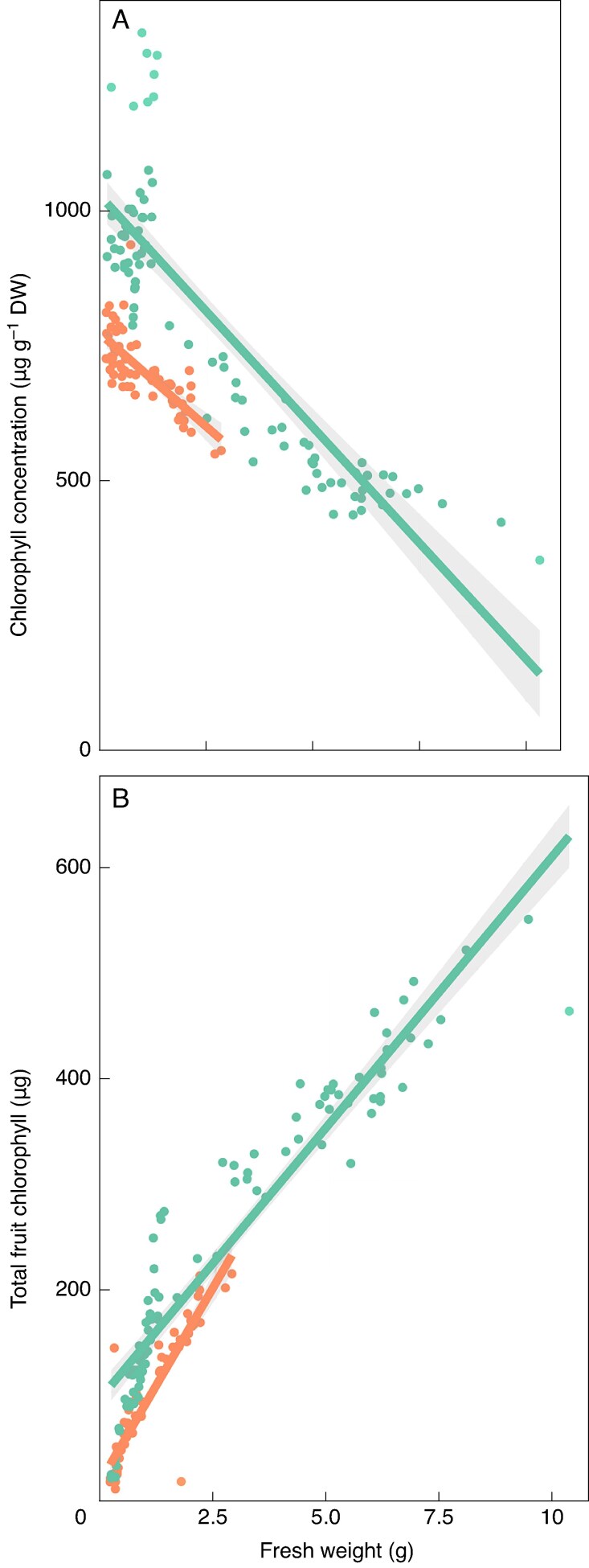
‘Fuji’ (teal) and ‘Honeycrisp’ (orange) linear regression lines between fresh weight (g) of chlorophyll concentration (A; µg g^−1^ DW) and total chlorophyll content (B; µg DW) of apple fruit sampled in North Carolina, USA, in 2023. Shaded area is the 95 % confidence interval of the regression line. Chlorophyll concentration and fresh weight: *r*^2^ = 0.756, *P* < 0.001 (‘Fuji’); *r*^2^ = 0.557, *P* < 0.001 (‘Honeycrisp’). Total chlorophyll content and fresh weight: *r*^2^ = 0.89, *P* < 0.001 (‘Fuji’); *r*^2^ = 0.822, *P* < 0.001 (‘Honeycrisp’). *N* = 90 (‘Fuji’); *N* = 65 (‘Honeycrisp’).

Chlorophyll concentration was negatively related to fruit size [‘Fuji’: *r*^2^ = 0.756, *P* < 0.001; ‘Honeycrisp’: *r*^2^ = 0.557, *P* < 0.001 (Fig. 6A)]. Among common fruit sizes (<2.86 g), ‘Fuji’ had higher chlorophyll concentration than ‘Honeycrisp’. The rate of decrease of chlorophyll concentration in this fruit weight range did not differ between cultivars (*P* = 0.947). However, the role of chlorophyll *a* and *b* differed between cultivars. With ‘Fuji’, the correlation coefficients between chlorophyll *a* or *b* and fruit fresh weight, CCI and PWI were similar (−0.868, *P* < 0.001 vs. −0.871, *P* < 0.001; −0.433, *P* < 0.001 vs. −0.429, *P* < 0.001; 0.673, *P* < 0.001 vs. 0.672, *P* < 0.001, respectively). For ‘Honeycrisp’, chlorophyll *a* showed greater correlation coefficient with these three same measures than chlorophyll *b* (−0.791, *P* < 0.001 vs. −0.524, *P* < 0.001; −0.463, *P* < 0.001 vs. −0.233, *P* = 0.062; 0.648, *P* < 0.001 vs. 0.356, *P* < 0.01, respectively).

Total fruit chlorophyll content was positively related to fruit weight [‘Fuji’: *r*^2^ = 0.89, *P* < 0.001; ‘Honeycrisp’: *r*^2^ = 0.822, *P* < 0.001 (Fig. 6B)]. Similar to the trends with chlorophyll concentration, ‘Fuji’ showed a greater total chlorophyll content than ‘Honeycrisp’ when fruit size was <2.86 g. The rate of increase in total chlorophyll with fruit fresh weight differed between cultivars (*P* < 0.001). The correlation between total chlorophyll and CCI or PWI was similar between cultivars. For CCI, this correlation coefficient was 0.575 (*P* < 0.001) and 0.565 (*P* < 0.001) for ‘Fuji’ and ‘Honeycrisp’, respectively. With PWI this correlation coefficient was −0.741 (*P* < 0.001) and −0.764 (*P* < 0.001) for ‘Fuji’ and ‘Honeycrisp’, respectively (Table 1).

**Fig. 7.**
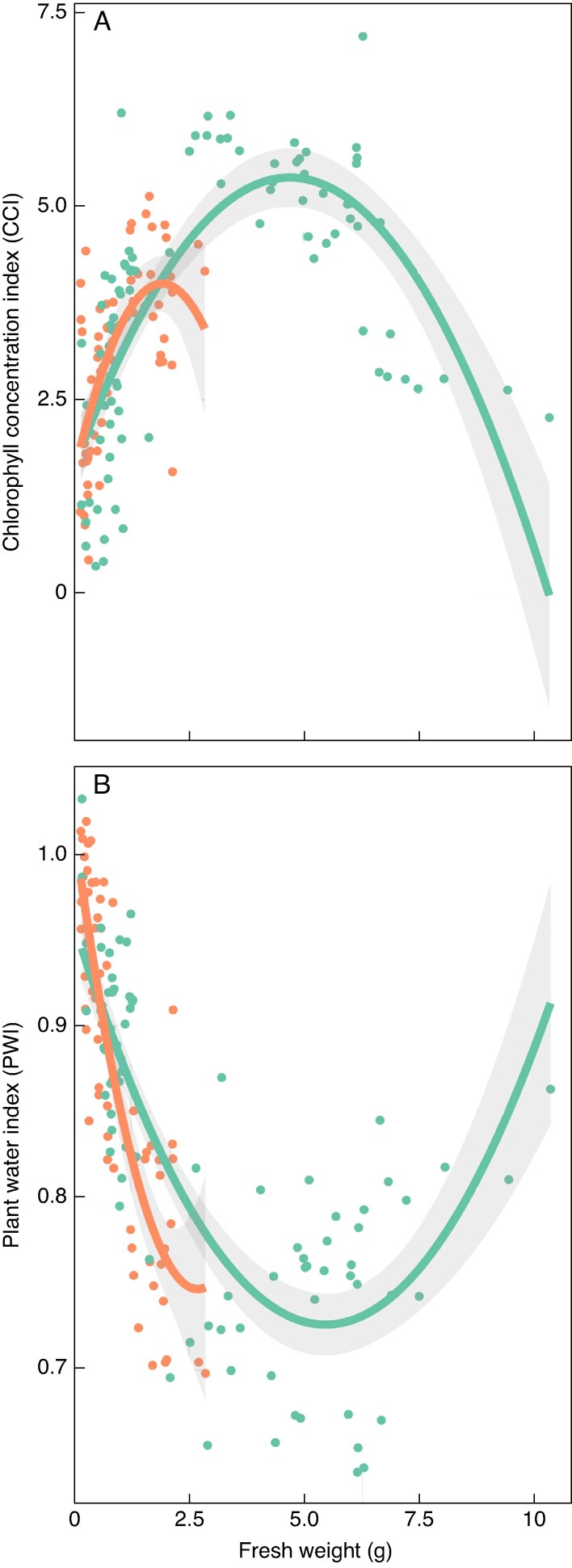
Second-order polynomial regression between fruit fresh weight (g) and chlorophyll concentration index (CCI, a.u.; A) or plant water index (PWI, a.u.; B) of ‘Fuji’ (teal) and ‘Honeycrisp’ (orange) apples sampled in 2023 in North Carolina, USA. Shaded area is the 95 % confidence interval of the regression line. Chlorophyll concentration index and fresh weight: *r*^2^ = 0.574, *P* < 0.001 (‘Fuji’); *r*^2^ = 0.426, *P* < 0.001 (‘Honeyrisp’). Plant water index and fresh weight: *r*^2^ = 0.697, *P* < 0.001 (‘Fuji’); *r*^2^ = 0.717, *P* < 0.001 (‘Honeyrisp’). *N* = 90 (‘Fuji’); *N* = 65 (‘Honeycrisp’).

There was a negative parabolic relationship between CCI and fruit fresh weight [‘Fuji’: *r*^2^ = 0.574, *P* < 0.001; ‘Honeycrisp’: *r*^2^ = 0.426, *P* < 0.001 (Fig. 7A)]. This relationship showed maxima at ∼2.0 and ∼5.0 g for ‘Honeycrisp’ and ‘Fuji’, respectively. The linear model explained less of the variation in the relationship between CCI and weight (‘Fuji’: *r*^2^ = 0.179, *P* < 0.001; ‘Honeycrisp’: *r*^2^ = 0.349, *P* < 0.001). Total chlorophyll content had a greater correlation coefficient with CCI (‘Fuji’: 0.575, *P* < 0.001; ‘Honeycrisp: 0.565, *P* < 0.001) than chlorophyll concentration [‘Fuji’: −0.432, *P* < 0.001; ‘Honeycrisp: −0.419 *P* < 0.001 (Table 1)].

### Water content and plant water index

Percentage water content of fruit stayed fairly constant at ∼85 % regardless of fruit size [‘Fuji’: *r*^2^ = 0.155 (*P* < 0.001); ‘Honeycrisp’: *r*^2^ = 0.021 (*P* = 0.249)]. Accordingly, there was not a strong correlation between percentage water content and CCI or PWI (Table 1). For ‘Fuji’ this correlation coefficient was 0.16 (*P* = 0.132) and −0.258 (*P* < 0.05) for CCI and PWI, respectively. The correlation coefficient between percentage water content and CCI for ‘Honeycrisp’ was 0.127 (*P* = 0.312) and −0.09 (*P* = 0.476) for PWI.

There was a parabolic relationship between PWI and fruit fresh weight [‘Fuji’: *r*^2^ = 0.697, *P* < 0.001; ‘Honeycrisp’: *r*^2^ = 0.717, *P* < 0.001 (Fig. 7B)] with minimum values at ∼2.5 and ∼5.0 g for ‘Honeycrisp’ and ‘Fuji’, respectively. These minimum values corresponded with the maxima of the parabolic relationship between CCI and fruit fresh weight. Comparatively, the linear model resulted in *r*^2^ values of 0.441 (*P* < 0.001) and 0.683 (*P* < 0.001).

## DISCUSSION

Of all the physiological characteristics measured, fruit size had the greatest implication on reflectance spectra of apple fruit early in development. Reflectance was lower on the second measurement date for both cultivars compared to the first collection date in two spectral regions: 550–700 nm and at ∼960 nm (Fig. 3). Fruit size also affected reflectance in the 775–900 nm NIR region as it was greater for fruit measured on the second collection dates than the first for both cultivars (Fig. 3). These findings align with previous results that more advanced fruit have lower reflectance in these two regions (Larson and Kon, 2023). Furthermore, there is a clear gradient of fruit size along PC1 that accounted for 86.56 % of the variance in the reflectance data (Fig. 4).

Both chlorophyll and trichome density related to fruit size. Chlorophyll concentration decreased, while total chlorophyll in the fruit increased as fruit grew (Fig. 6). The visual observations of Orlova *et al*. (2020*b*) that trichome density is lowest at the centre of the fruit, higher at the calyx or pedicel end, and that trichome density decreases with fruit size were confirmed in this study (Fig. 5). Among the three positions of the fruit where trichome density was examined, the calyx end had the strongest correlation with fruit size than the centre or pedicel end of the fruit. More of the variance in total chlorophyll content was explained by fruit size (‘Fuji’: *r*^2^ = 0.89, *P* < 0.001; ‘Honeycrisp’: *r*^2^ = 0.822 *P* < 0.001) than trichome density at the calyx end of the fruit (‘Fuji’: *r*^2^ = 0.608, *P* < 0.001; ‘Honeycrisp’: *r*^2^ = 0.586, *P* < 0.001).

Considering the impact of fruit size on reflectance spectra, chlorophyll content has a greater effect on reflectance than trichome density. This is confirmed by there being only one case – ‘Honeycrisp’ sampled 20 DAFB – in the current study were the removal of trichomes shifted reflectance spectra (Fig. 3). There, reflectance was higher once trichomes were removed from ∼525 to 1098 nm. This is the opposite finding of Orlova *et al*. (2020*b*) who found lower reflectance with trichome removal and concluded that trichome density was the driving factor in lower light reflectance of larger fruit. The method of trichome removal may explain the differences in this trend between these two studies. Orlova *et al*. (2020*b*) removed trichomes with an optical cloth while the current study employed tape. Removal with tape is more likely to remove wax along with the trichomes than would rubbing off alone. This would indicate that wax deposits on the surface of fruit play a larger role in reflectance spectroscopy than trichomes.

Beyond trichomes, waxes and chlorophyll, anthocyanins are also present on the surface of fruit at the stage of development focused on in this study. Larson *et al*. (2023) found that there were no differences in anthocyanin concentration between fruitlets that were predicted to either persist or abscise with reflectance spectroscopy models that relied on the same wavelengths used in the current study. During the 10 d span that fruitlets were measured, anthocyanin concentration stayed relatively constant (Larson *et al*., 2023). Based on those results we did not quantify anthocyanin concentration in the current study. Anthocyanins do vary throughout the growing season. Understanding these variations throughout fruit development and its effects on light reflectance warrants further study to provide a clear picture of physiological characteristics and reflectance spectroscopy throughout the growing season.

Fruit size also played a role in the CCI and PWI. For each of these indices there was a maximum (CCI) or minimum (PWI) fruit weight after which the indices decreased (CCI) or increased (PWI) to values observed for smaller fruit (Fig. 7). This trend is probably due to the portable spectrometer used in the study. This spectrometer has lens with a fixed size (∼30 mm in diameter) and the amount of light sent by the spectrometer is constant. As fruit grow, more of the lens is covered by the fruit and more light can interact with the fruit. For example, a larger fruit will cover more of the lens and have more total chlorophyll to absorb more red-light. This fruit size effect reaches a maximum once the fruit entirely covers the lens. However, chlorophyll concentration decreases as fruit grow, so once a fruit occupies the entire lens the reflectance of red-light will only increase as the chlorophyll in that area becomes diluted. For ‘Fuji’ fruit, the point at which the fruit entirely covers the lens is ∼5.0 g, where CCI reaches a maximum. Other spectrometers that can measure entire fruit that are larger or computer vision systems would probably not experience this relationship. Therefore, reflectance spectroscopy-based models to predict chemical thinner efficacy are probably spectrometer-specific.

Differences in chlorophyll content among cultivars were revealed in the current study. At weights < 2.86 g, ‘Fuji’ had a higher chlorophyll concentration of ∼250 µg·g^−1^ DW than ‘Honeycrisp’ (Fig. 6). ‘Fuji’ also had greater total chlorophyll content than ‘Honeycrisp’ at the smallest fruit sizes sampled, but the rate of increase of total chlorophyll was greater for ‘Honeycrisp’ so that total chlorophyll was nearly equal between the two cultivars for the largest ‘Honeycrisp’ sampled [2.86 g (Fig. 6)]. These differences may be telling for the photosynthetic capabilities and the capacity for the fruit to produce carbohydrates to support growth when imported carbohydrates are limited early in fruit development. If this link does exist, it would correlate with the susceptibility of these cultivars to chemical thinners as ‘Fuji’ is less susceptible than ‘Honeycrisp’. The link between fruit chlorophyll content, photosynthetic capability and chemical thinner susceptibility merits further investigation and could answer long-held questions about fruit abscission mechanisms.

For ‘Fuji’ the correlations of chlorophyll *a* or *b* concentration with fruit size, CCI or PWI were similar; however, for ‘Honeycrisp’ there was a decrease in the correlations between chlorophyll *b* concentration and these three measures compared to their correlations with chlorophyll *a* concentration (Table 1). This was due to a slower decrease in chlorophyll *b* concentration as fruit expanded for ‘Honeycrisp’ compared to ‘Fuji’. When comparing the two cultivars at similar fruit weights, the interaction between fruit weight and cultivar was significant for the ratio of chlorophyll *a*/*b* (*P* < 0.001) but not chlorophyll *a* (*P* = 0.724) or chlorophyll *b* (*P* = 0.199. This indicates that the ratio of chlorophyll *a/b* changed for ‘Honeycrisp’ but not for ‘Fuji’. These results again illustrate the variability in fruit chlorophyll content among apple cultivars and these differences need further investigation.

Both Orlova *et al*. (2020*a*) and Larson and Kon (2023) have shown the utility in using reflectance spectroscopy to distinguish between persisting and abscising fruitlets following a chemical thinner application. Those studies found two key wavelength areas where persisting fruit reflect less light than abscising: red-light and at ∼975 nm. These results find that it is ultimately fruit size during this stage of development that is influencing the reflectance at these wavelengths that the developed models use to predict whether or not a fruitlet will abscise. Larger fruit have more total chlorophyll to absorb red-light and water to absorb light at ∼975 nm. This finding aligns with previous research that finds that large fruit that are the strongest sinks for carbohydrates are most likely to persist following a chemical thinner application (Ward and Marini, 1999; Greene *et al*., 2013). While this study found differences in total chlorophyll content between cultivars that may be important for the photosynthetic capacity of these fruit, red-light reflectance does not differ between cultivars on either sampling date. This means that this physiological difference is not likely to influence the capacity to predict chemical thinning efficacy with reflectance spectroscopy.

## CONCLUSIONS

Fruit size had the greatest effect on reflectance spectra of young apple fruit. Larger fruit resulted in lower reflectance in the red-light and water absorption (∼975 nm) bands of the spectrum. As fruit expanded, chlorophyll concentration and trichome density decreased, while total chlorophyll in the fruit increased. The relationship between fruit size and trichome density was strongest at the calyx end than at the centre or pedicel end of the fruit. Only for ‘Honeycrisp’ fruit sampled at 20 DAFB did trichome removal increase reflectance, and otherwise there were no differences in reflectance between fruit with or without trichomes. Chlorophyll also had a stronger relationship with fruit size than it did with trichome density. ‘Fuji’ fruit had greater total chlorophyll than ‘Honeycrisp’ at similar fruit sizes. The role of fruit chlorophyll content and photosynthetic capacity merits further investigation, particularly in the context of chemical thinning. Elucidating how far light at different wavelengths penetrates into young apple fruitlets will further answer how reflectance spectra vary with growth stage and among different cultivars.

## References

[mcaf124-B1] Blanke MM, Lenz F. 1989. Fruit photosynthesis. Plant, Cell & Environment 12: 31–46. doi:10.1111/j.1365-3040.1989.tb01914.x

[mcaf124-B2] Burns BW, Green VS, Hashem AA , et al 2022. Determining nitrogen deficiencies for maize using various remote sensing indices. Precision Agriculture 23: 791–811. doi:10.1007/s11119-021-09861-4

[mcaf124-B3] Byers RE, Barden JA, Polomski RF, Young RW, Carbaugh DH. 1990. Apple thinning by photosynthetic inhibition. Journal of American Society of Horticultural Sciences 115: 14–19. doi:10.21273/JASHS.115.1.14

[mcaf124-B4] Dias PA, Tabb A, Medeiros H. 2018. Multispecies fruit flower detection using a refined semantic segmentation network. IEEE Robotics and Automation Letters 3: 3003–3010. doi:10.1109/LRA.2018.2849498

[mcaf124-B5] Faust M, Shear CB. 1972. Fine structure of the fruit surface of three apple cultivars. Journal of the American Society of Horticultural Sciences 97: 351–355. doi:10.21273/JASHS.97.3.351

[mcaf124-B6] Gitelson AA, Gritz Y, Merzlyak MN. 2003. Relationships between leaf chlorophyll content and spectral reflectance and algorithms for non-destructive chlorophyll assessment in higher plant leaves. Journal of Plant Physiology 160: 271–282. doi:10.1078/0176-1617-00887 12749084

[mcaf124-B7] Gonzalez Nieto L, Wallis A, Clements J , et al 2023. Computer vision systems and applications to estimate trunk cross-sectional area, flower cluster number, thinning efficacy and yield of apple. Horticulturae 9: 880. doi:10.3390/horticulturae9080880

[mcaf124-B8] Govender M, Dye PJ, Weiersbye IM, Witkowski ETF, Ahmed F. 2009. Review of commonly used remote sensing and ground-based technologies to measure plant water stress. WaterSA 35: 741–752. doi:10.4314/wsa.v35i5.49201

[mcaf124-B9] Greene DW, Lakso AN, Robinson TL, Schwallier P. 2013. Development of a fruitlet growth model to predict thinner response on apples. HortScience 48: 584–587. doi:10.21273/HORTSCI.48.5.584

[mcaf124-B10] Ji W, Zhao D, Cheng F, Xu B, Zhang Y, Wang J. 2012. Automatic recognition vision system guided for apple harvesting robot. Computers & Electrical Engineering 38: 1186–1195. doi:10.1016/j.compeleceng.2011.11.005

[mcaf124-B11] Jing S, Malladi A. 2020. Higher growth of the apple (*Malus domestica* Bork l.) fruit cortex is supported by resource intensive metabolism during early development. BMC Plant Biology 20: 75. doi:10.1186/s12870-020-2280-2 32054442 PMC7020378

[mcaf124-B12] Konarska A . 2014. Differences in the structure of fruit buds in two apple cultivars with particular emphasis on features responsible for fruit storability and quality. Acta Scientiarum Polonorum Hortorum Cultus 13: 91–105.

[mcaf124-B13] Lakso AN, Wünsche JN, Palmer JW, Grappadelli LC. 1999. Measurement and modeling of carbon balance of the apple tree. HortScience 34: 1040–1047. doi:10.21273/HORTSCI.34.6.1040

[mcaf124-B14] Lammertyn J, Peirs A, De Baerdemaeker J, Nicolaï B. 2000. Light penetration properties of NIR radiation in fruit with respect to non-destructive quality assessment. Postharvest Biology and Technology 18: 121–132. doi:10.1016/S0925-5214(99)00071-X

[mcaf124-B15] Larson JE, Kon TM. 2023. Apple fruit abscission prediction. I. Development and evaluation of reflectance spectroscopy models. HortScience 58: 1085–1092. doi:10.21273/HORTSCI17244-23

[mcaf124-B16] Larson JE, Kon TM, Malladi A. 2022. Apple fruitlet abscission mechanisms. In: Warrington I. ed. Horticultural reviews. Chichester: Wiley, 243–274.

[mcaf124-B17] Larson JE, Perkins-Veazie P, Kon TM. 2023. Apple fruit abscission prediction. II. Characteristics of fruits predicted to abscise or persist by reflectance spectroscopy models. HortScience 58: 1095–1103. doi:10.21273/HORTSCI17245-23

[mcaf124-B18] Merzlyak MN, Solovchenko AE, Gitelson AA. 2003. Reflectance spectral features and non-destructive estimation of chlorophyll, carotenoid, and anthocyanin content in apple fruit. Postharvest Biology and Technology 27: 197–211. doi:10.1016/S0925-5214(02)00066-2

[mcaf124-B19] Orlova Y, Linker R, Spektor B. 2020a. Forecasting the potential of apple fruitlet drop by in-situ Vis-NIR spectroscopy. Computers and Electronics in Agriculture 169: 105225. doi:10.1016/j.compag.2020.105225

[mcaf124-B20] Orlova Y, Linker R, Spektor B. 2020b. Selection of Vis-NIR wavebands for forecasting apple fruitlet drop in response to chemical treatment. Biosystems Engineering 195: 172–185. doi:10.1016/j.biosystemseng.2020.05.001

[mcaf124-B21] Peñuelas J, Filella I, Biel C, Serrano L, Save R. 1993. The reflectance at the 950-970 nm region as an indicator of plant water status. International Journal of Remote Sensing 14: 1887–1905.

[mcaf124-B22] Populin F, Pellizzari P, Costa G, Meggio F, Botton A. 2022. Thermographic imaging to identify abscising apple fruitlets after a thinning treatment. In: EUFRIN Fruit Thinning Working Group Symposium, Belgrade, Serbia. ISHS Acta Horticulturae, 1341: 56–54.

[mcaf124-B23] Prasad AK, Chai L, Singh RP, Kafatos M. 2006. Crop yield estimation model for Iowa using remote sensing and surface parameters. International Journal of Applied Earth Observation and Geoinformation 8: 26–33. doi:10.1016/j.jag.2005.06.002

[mcaf124-B24] Pratt C . 1988. Apple flower and fruit: morphology and anatomy. In: Janick J. ed. Horticultural reviews. Portland, OR: Timber Press, 273–308.

[mcaf124-B25] R Core Team . 2022. R: a language and environment for statistical computing. Vienna: R Foundation for Statistical Computing.

[mcaf124-B26] Sims DA, Gamon JA. 2002. Relationships between leaf pigment content and spectral reflectance across a wide range of species, leaf structures and developmental stages. Remote Sensing of Environment 81: 337–354. doi:10.1016/S0034-4257(02)00010-X

[mcaf124-B27] Sun K, Wang X, Liu S, Liu C. 2021. Apple, peach, and pear flower detection using semantic segmentation network and shape constraint level set. Computers and Electronics in Agriculture 185: 106150. doi:10.1016/j.compag.2021.106150

[mcaf124-B28] Ward D, Marini RP. 1999. Growth and development of young apple fruits following applications of ethephon plus carbaryl for thinning. HortScience 34: 1057–1059. doi:10.21273/HORTSCI.34.6.1057

[mcaf124-B29] Watt MS, Pearse GD, Dash JP, Melia N, Leonardo EMC. 2019. Application of remote sensing technologies to identify impacts of nutritional deficiencies on forests. ISPRS Journal of Photogrammetry and Remote Sensing 149: 226–241. doi:10.1016/j.isprsjprs.2019.01.009

[mcaf124-B30] Wood CW, Reeves DW, Himelrick DG. 1993. Relationships between chlorophyll meter readings and leaf chlorophyll concentration, N status, and crop yield: a review. Proceedings Agronomy Society of N.Z. 23: 1–9.

[mcaf124-B31] Wrolstad RE, Durst RW, Lee J. 2005. Tracking color and pigment changes in anthocyanin products. Trends in Food Science & Technology 16: 423–428. doi:10.1016/j.tifs.2005.03.019

